# Enhanced Oligopeptide and Free Tryptophan Release from Chickpea and Lentil Proteins: A Comparative Study of Enzymatic Modification with Bromelain, Ficin, and Papain

**DOI:** 10.3390/plants13213100

**Published:** 2024-11-03

**Authors:** Éva Domokos-Szabolcsy, Tarek Alshaal, Nevien Elhawat, Zoltán Kovács, László Kaszás, Áron Béni, Attila Kiss

**Affiliations:** 1 Department of Applied Plant Biology, Faculty of Agricultural and Food Sciences and Environmental Management, University of Debrecen, Böszörményi Str. 138, 4032 Debrecen, Hungary; szabolcsy@agr.unideb.hu (É.D.-S.); elhawat.nevien@agr.unideb.hu (N.E.); kovacs.zoltan@agr.unideb.hu (Z.K.); kaszas.laszlo@agr.unideb.hu (L.K.); 2 Soil and Water Science Department, Faculty of Agriculture, Kafrelsheikh University, Kafr El-Sheikh 33516, Egypt; 3 Faculty of Agriculture (for Girls), Al-Azhar University, Tanta 31732, Egypt; 4 Institute of Agricultural Chemistry and Soil Science, Faculty of Agricultural and Food Sciences and Environmental Management, University of Debrecen, Böszörményi Str. 138, 4032 Debrecen, Hungary; beniaron@agr.unideb.hu; 5 Agro- and Food-Industrial Innovation Centre, University of Debrecen, Böszörményi Str. 138, 4032 Debrecen, Hungary; attkiss@agr.unideb.hu

**Keywords:** plant-based proteases, degree of hydrolysis, free amino acids, SDS-PAGE analysis, protein hydrolysis

## Abstract

Plant-based foods offer a sustainable alternative to meet the growing protein demand. Legumes are the most promising of these, as they contain relatively high concentrations of protein, low digestible starch, and dietary fiber, as well as them possibly featuring low levels of fat. Enzymatically modified legume proteins provide us with tempting perspectives in terms of enhancing foods’ biological values. However, their bioavailability and digestibility are generally less sufficient than that of proteins of animal origin, which may be improved by well-tailored enzyme modification. In this study, the efficacy of three plant-based proteases (bromelain, ficin, and papain) were evaluated at two distinct concentrations (2.5% and 10%) and three hydrolysis durations (1, 2, and 12 h) when transforming chickpea and lentil proteins. The degree of hydrolysis (DH), peptide profiles, and free amino acid content were analyzed to determine the efficiency of each enzyme. Results showed significant variations in DH, which was influenced by enzyme type, concentration, and hydrolysis duration. Papain exhibited the highest DH, particularly at a 10% concentration, reaching 27.8% efficiency in chickpea and 34.8% in lentils after 12 h. Bromelain and ficin were proven to be less effective, with ficin showing the least hydrolytic activity. SDS-PAGE analysis revealed substantial protein degradation, especially subsequent to papain treatment, pointing out that most proteins were cleaved into smaller peptides. SEC-HPLC indicated a predominant release of peptides within the 200–1000 Da range, suggesting enhanced bioavailability. Papain and bromelain treatments resulted in a significant release of oligopeptides and dipeptides. UHPLC analysis highlighted a marked post-hydrolysis increase in total free amino acids, with arginine, leucine, and lysine being the most abundant ones. Notably, tryptophan, being undetectable in untreated samples, was released in measurable amounts post-hydrolysis. These findings demonstrate papain’s superior performance in protein hydrolysis and its potential in producing bioactive peptides, highlighting its applicability in food processing and the development of both nutraceuticals and functional foods.

## 1. Introduction

Plant-based proteins and the revealing of novel protein sources are increasingly necessary to provide alternatives to traditional animal-derived foods. Factors such as the growing global population, rising protein demand, and concerns about the environmental impact, ethics, and animal health, as well as issues concerning the security of animal-based foodstuffs, highlight the need for the production of foods of increased protein content and improved bioavailability and bioutilization [1,2]. The definite shift in consumers’ attitudes towards the preference of plant-based foods underpins the significance of relevant research activities.

The direct consumption of plant proteins, particularly pulses like peas, beans, chickpeas, lentils, and lupins, is more efficient and sustainable than using these crops for animal feed [3,4]. Pulses, with a protein content of 15–30% of the dry matter [5], are being recognized as nutritious and sustainable protein sources. However, producing high-protein ingredients often requires concentration or isolation processes. Dry processing methods, such as milling and air classifying, can yield protein concentrates with up to 70% protein content, depending on the pulse [6,7].

When developing plant-based products, there is often a discrepancy between the desired functionality and the actual features, as well as the health benefits of the implied protein ingredients. Pulse proteins, in particular, have poor solubility under mildly acidic conditions near their isoelectric point due to electrostatic repulsion [8]. Partial enzymatic hydrolysis has been shown to improve the solubility, techno-functional properties, and biological value of proteins with initially poor functionality [9]. Additionally, hydrolysis can enhance digestibility, as was demonstrated with lentil proteins employed in in vitro experiments [10]. Enzymatic hydrolysis is often preferred over chemical hydrolysis as it requires milder conditions, is easier to control, and preserves the nutritional quality of the protein [11]. This process is crucial in the food industry to improve proteins’ physiologically beneficial properties and increase the solubility as well as techno-functional characteristics, and also to reduce allergenicity. Hydrolysis alters protein structure, breaking them into peptides and amino acids, and modifies the amino acid profile, which impacts protein allergenicity at the level of IgE binding, an antibody involved in allergic reactions [12].

Proteases, or peptidases, are enzymes that break down peptides and proteins through hydrolysis. They are classified based on the positional specificity of endopeptidases, which cleave internal polypeptide bonds, and exopeptidases, which cleave near the C- or N-terminus, producing single amino acids, dipeptides, or tripeptides [13]. Proteases are also categorized by the main chemical group at their catalytic site, including serine, cysteine, threonine, aspartic acid, glutamic acid, and metalloproteases [13]. Additionally, they can be classified by regarding their origin: microbial, plant, or animal-derived [14]. Among these, a few numbers of animal-derived proteases are used on an industrial scale, including rennin, trypsin, and pepsin. The extraction of animal proteases is affected by the slaughter of livestock. This extraction is regulated by different policies. At the same time, the use of animal proteases, and with it, consumer acceptance, is limited by the potential risk of disease transmission, environmental concerns, sustainability, animal welfare, and ethical issues [13]. By contrast, plant proteases are of growing interest, especially in food applications. Plant proteases are considered readily available; they are generally more cost-effective than other proteases, typically have high proteolytic activity, and are uniquely stable over a higher temperature range, allowing a more controlled process without destroying essential amino acids [15]. Food-grade proteases, especially endoproteases, are commonly used to produce pulse protein hydrolysates, often in combination with exoproteases. While commercial enzyme preparations may contain single or mixed proteases, their application has largely been limited to soybean and milk proteins so far. However, the rising demand for plant-based proteins has spurred a growing interest in legume proteins from chickpeas, beans, lentils, and peas, though research activities on their bioavailability, digestibility, and antioxidant activities, as well as their degradation perspectives, remain limited [16,17].

Plant proteases play vital roles throughout the life cycle of plants, especially during germination by mobilizing seed and grain proteins. Cysteine proteases, including papain-like C1A and legumains (C13), are key players in these processes [18,19]. They also assist in programmed cell death, senescence, nutrient remobilization under stress, and giving responses to pathogens [20,21]. Industrially, proteases like papain have been utilized in the food, pharmaceutical, detergent, and biofuel industries for decades [22]. New plant proteases might be regarded as promising candidates for food industrial applications, such as breaking down gluten peptides that are harmful to celiac patients [23].

Plant proteases, unlike bacterial and fungal peptidases, are natural components of food and generally do not require safety validation, making them convenient for therapeutic and food processing applications [24]. For instance, some previous research [25] demonstrated the antioxidant potential and thermal stability of chickpea proteins, particularly those rich in albumin. In subsequent studies, it was found that hydrolyzing chickpea globulins with alcalase and pepsin significantly improved their antioxidant properties, solubility, and digestibility [26].

The selection of papain, ficin, and bromelain for preparing protein hydrolysates from chickpeas and lentils in the present study is based on their distinct proteolytic properties and their ability to break down complex plant proteins into bioactive peptides and amino acids. Papain, from Carica papaya, is used in meat, dairy, baking, brewing, wine, and bioethanol industries, as well as in biomedical and oral care for its proteolytic and antibacterial properties. It is stable under a wide range of temperatures and pH levels, which makes it a robust choice for protein hydrolysis in various environments [27].

Bromelain, from pineapple, is used in meat, fish, alcoholic beverage, animal feed, and textile industries due to its protein solubilization abilities [28]. It is known for its strong proteolytic activity and ability to break down a wide range of protein substrates, including plant proteins like those in chickpeas and lentils. Similarly to papain, bromelain is stable under different conditions, making it suitable for both experimental and industrial applications [29].

One of the most promising plant-derived proteases is ficin, which is found in the latex of figs (*Ficus carica*). It belongs to the group of cysteine (Cys) endopeptidases that include papain, bromelain, calpain, cathepsin B, and chymopapain [30]. Ficin from fig latex is characterized as a mixture of cysteine proteases functionally related to papain. Ficin can produce peptides that are smaller or have particular bioactive properties, which is often the goal of protein hydrolysis in food and bioactive peptide research. Similarly to papain and bromelain, ficin has a broad pH stability range and is particularly effective in hydrolyzing plant proteins [31].

Papain, ficin, and bromelain are all derived from common plant sources (papaya, figs, and pineapples), and their extraction is well established, making them accessible for industrial use. Advances in enzyme production techniques, such as microbial fermentation, have further improved the scalability and reduced the cost of these enzymes [27]. Due to their high efficiency in breaking down proteins, only small amounts of these enzymes are required to achieve significant hydrolysis, which reduces the overall enzyme cost in large-scale operations. This efficiency contributes to their feasibility for industrial-scale use in hydrolyzing plant materials like chickpeas and lentils. Industrial-grade enzymes are often sold in bulk, making them more economically viable. Enzyme activity (measured in units) plays a significant role in determining the cost. Highly active enzymes are more expensive but are also more efficient, requiring less material for hydrolysis [32,33].

The aim of this study was to validate a practical method for producing protein hydrolysates from legume seeds, specifically chickpea and lentil, utilizing plant-derived proteases such as papain, bromelain, and ficin. The validation of this method is based on the assessment of the degree of hydrolysis, amino acid composition, oligo- and polypeptide profiles, and protein patterns.

## 2. Materials and Methods

### 2.1. Materials

Seeds of two plant species, chickpea (*Cicer arietinum*) and lentil (*Lens culinaris*), were included in the present experimental work. The dry seeds were purchased from the market as commercial products. Three plant-based cysteine proteases were applied for the hydrolysis: bromelain with an activity of 3 U/mg (Merck Life Science, Darmstadt, Germany), papain with an activity of 1000 TU/mg activity (Thermo Fisher Scientific, Waltham, MA, USA), and ficin with an activity of 600 MCU/mg (Thermo Fisher Scientific, Waltham, MA, USA).

### 2.2. Determination of Total Protein Content

The total protein content in powdered chickpea and lentil flour was measured by determining the total nitrogen content using the Dumas method with an Elementar Vario Max Cube (Elementar Analysensysteme GmbH, Langenselbold, Germany) [34].

### 2.3. Preparation of Protein Hydrolysates

Chickpea and lentil flour was suspended in 2% NaCl solution at a ratio of 1:35 (*m*/*v*) and incubated at 37 °C for 1 h on a rotary shaker set to 120 rpm to recover soluble proteins (e.g., globulins and albumins). The pH of the mixture was adjusted to 7 for bromelain and ficin and to 8 for papain [30,35]. The mixture was then inoculated with the enzyme in ratios of 2.5% (975 mg sample + 25 mg enzyme) and 10%, (900 mg sample + 100 mg enzyme) and incubated for 1, 2, and 12 h in a thermostatically controlled cabinet at 50 °C on an oscillating shaker at 120 rpm. After hydrolysis, the mixture was heated to 90 °C for 5 min to inactivate the enzymes. The hydrolyzed samples were subsequently freeze-dried using a lyophilizer (Alpha Christ, Osterode am Harz, Germany).

### 2.4. Degree of Hydrolysis

The degree of hydrolysis (DH) is an important parameter to feature the extent of protein breakdown and can be quantified using various methods. In the present study, the DH of protein hydrolysates was quantified by the ortho-phthaldialdehyde (OPA) method, one of the most widely used methods, which detects the free amino groups released during hydrolysis. Briefly, a 0.1 g lyophilized protein hydrolysate sample containing 8% to 80% crude protein was dissolved in 100 mL of distilled water. After vortex for 2 min, samples were placed in an ultrasonic bath for 20 min to facilitate dissolving the hydrolyzed amino acids. In a 10 mL test tube containing 3 mL OPA reagent (200 mL OPA reagent containing 7.620 g disodium tetraborate + 0.20 g sodium dodecyl sulfate + 0.16 g OPA dissolved in 4 mL ethanol (96%) + 0.176 g dithiothreitol), 400 µL of the sample was added. Both blank and standard samples were treated in the same way as the measured sample using 400 µL distilled water and L-serine (0.9516 meq/L), respectively, instead of the sample. Samples were shaken and absorbance was measured at 340 nm using the model UV-160A spectrophotometer (Shimadzu, Kyoto, Japan). All samples were examined in three repeats. The DH was calculated as follows [36]:

$$DH,\%=\frac{h}{h(tot)}*100$$

where *h*(*tot*) = total amino acid content, which equals 8 for the non-examined materials, and *h* = free amino acids, which is calculated as follows:

$$h=\frac{\left(Serine\text{-}NH_{2}-\beta \right)}{\alpha }$$

where *β* = 0.4 meq/g protein (constant of the non-examined materials), *α* = 1.0 meq/g protein (constant of the non-examined materials), and *Serine-NH*_2_ = meq *serine-NH*_2_/g protein, which can be calculated as follows:

$$Serine\text{-}NH_{2}=\frac{\left(Abs\;sample-Abs\;blank\right)*0.9516*sample\;volume\left(L\right)*100}{\left(Abs\;standard-Abs\;blank\right)*sample\;weight\left(g\right)*protein\;\%}$$

where 0.9516 = concentration of L-serine standard (meq/L).

### 2.5. Protein Pattern by SDS-PAGE

The protein expression patterns of untreated and hydrolyzed chickpea and lentil seed samples were evaluated by sodium dodecyl sulfate–polyacrylamide gel electrophoresis (SDS-PAGE). For sample preparation, 10 mg of the freeze-dried sample and 250 uL of solubilization buffer (4× Laemmli buffer) were mixed thoroughly via vortex. The samples were incubated for 5 min at 95 °C in a block heater (Eppendorf ThermoStat Plus Block Heater). Then, they were centrifuged at 13,000 rpm for 20 min at 4 °C. The supernatant was used for further analysis. SDS-PAGE was performed in a vertical system on a discontinuous polyacrylamide gel. A 12.5% resolving gel was prepared; the stacking gel was 9%. The molecular weight separation of soluble proteins was carried out in a Mini-Protean Tetra Cell gel system (Bio-Rad Inc., Hercules, CA USA) at 90 V for 10 min and then continued at 180 V for ~50 min. Gels were stained with Coomassie G250 staining solution and analyzed using the BioRad ChemiDoc MP Imaging System.

### 2.6. Peptides Separation by SEC-HPLC

#### 2.6.1. Sample Preparation

A 50 mg powdered sample was mixed with 5 mL of 1% phosphate-buffered saline (PBS) buffer. The samples were incubated in an ultrasonic bath for 60 min and centrifuged at 5000 rpm for 5 min. Before separation, samples were filtered through a 0.22 µm PTFE filter.

#### 2.6.2. Measurements

An ECOM liquid chromatograph consisting of a photodiode array (PDA) detector (model ECDA) (ECOM, Prague, Czech Republic) was used to separate peptides/oligopeptides. Separations were performed on a Agilent AdvanceBio SEC column (300 Å; 4.6 × 300 mm × 2.7 μm) using an isocratic elution system; the mobile phase consisted of 1% PBS buffer. The flow rate was maintained at 0.7 mL/min. The detection wavelength was 280 nm. Two different standards were used to measure the molecular weight and concentration of peptides by SEC analysis. One is the Bio-Rad gel filtration standard (Bio-Rad, Hercules, USA), ranging from 1350 to 670,000 Da, containing a thyroglobulin (MW: 670,000 Da), g-globulin (MW: 158,000 Da), ovalbumin (MW: 44,000 Da), myoglobin (MW: 17,000 Da) and vitamin B12 mixture (MW: 1350 Da). The other peptide standard mixture (Merck, Rahway, NJ, USA) contains Gly-Tyr (MW: 238.2 Da), Val-Tyr-Val (MW: 379.5 Da), methionine encephalin (MW: 573.7 Da), leucine encephalin (MW: 555.6 Da), and angiotensin II peptides (MW: 1046.2 Da). The molecular weight in Da units is calculated by the Dataapex Clarity software (GPC module) using the following equation with a correlation factor of 0.9984.

Y=0.3896X+8.461

### 2.7. Quantification of Free Amino Acid Profile

Prior to the quantification of the amino acid profiles of untreated and hydrolysed chickpea and lentil seed samples by ultra-high pressure chromatography (UHPLC) using a Waters Acquity UPLC System H class plus (Waters, Milford, MA, USA), a borate buffer solution, supplied by Waters Co., was employed to adjust the pH of the samples to 8, thereby establishing the optimal chemical conditions for the reaction. The 6-aminoquinolyl-N-hydroxysuccinimidyl carbamate (AQC) reagent was applied to react with both primary and secondary amines. The separation of free amino acids was based on a AccQTag pre-column derivatization procedure. Following the manufacturer’s instructions, the samples were derivatized applying AccQ-Tag Ultra derivatization reagent kit (Waters, Milford, MA, USA) [37]. The separation of the derivatized amino acids was performed by using AccQ-tag Ultra C18 column (1.7 µm; 2.1 × 100 mm, Waters, Milford, MA, USA) guarded by an Accquity in-line filter (0.2 µm; 2.1 mm, Waters, Milford, MA, USA). The flow rate was 0.100 mL/min, and a 54 °C column temperature was applied in gradient elution (11 min long gradient). A Quaternary gradient pump mixed solvent A, which was 100% AccQ-tag Ultra eluent A; solvent B, which was 10% AccQ-tag Ultra eluent B in LC–MS-grade water; solvent C, which was LC water; and solvent D, which was 100% AccQ-tag Ultra eluent B. Data were evaluated with the application of Waters Empower 3 software (Waters, Milford, MA, USA).

### 2.8. Statistical Analysis

The homogeneity of the dependent variables was verified. Data analysis was conducted by using Microsoft Excel 2016 and the SPSS 25.0 software package (SPSS Inc., Chicago, IL, USA). One-way ANOVA was employed to evaluate the differences among treatments. Mean separation was conducted using Tukey’s post hoc test, with significant differences recognized at *p* < 0.05. The results are presented as mean ± standard deviations.

## 3. Results and Discussion

### 3.1. Protein Expression Pattern by SDS-PAGE

**Figure 1:**
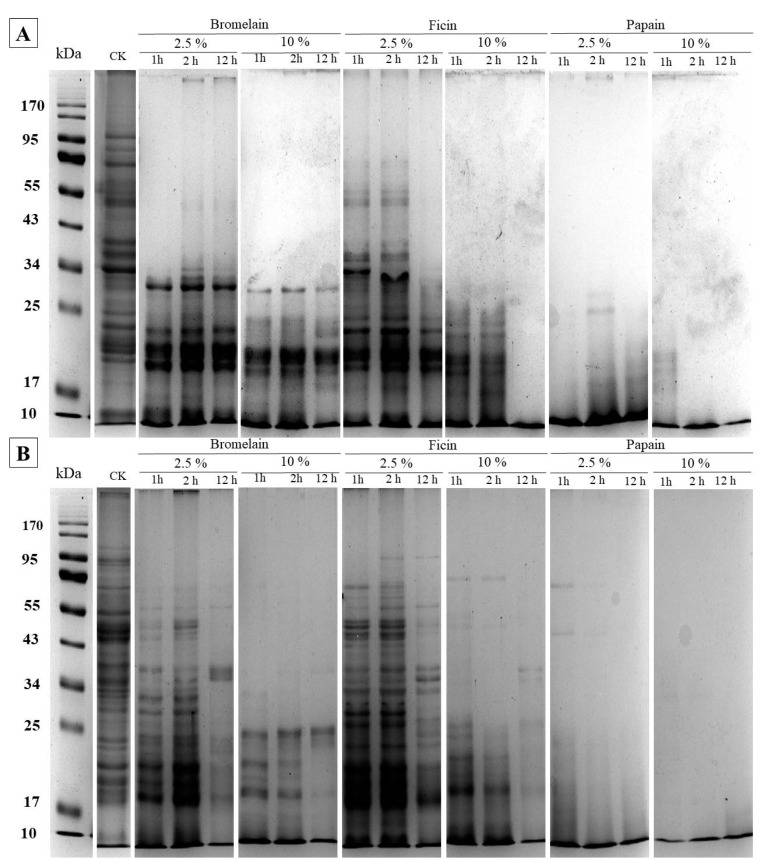
SDS-PAGE of (**A**) chickpea and (**B**) lentil seeds hydrolyzed by three plant-based proteases (i.e., bromelain, ficin, and papain) at two enzyme concentrations (2.5% and 10%) for different hydrolysis times (1, 2, and 12 h). CK = control (untreated seed).

SDS-PAGE analysis in this study provided valuable insights into the breakdown patterns of chickpea and lentil proteins following enzymatic hydrolysis with papain, bromelain, and ficin. The analysis allowed us to observe protein band changes at specific molecular weights, reflecting the degradation of high-molecular-weight proteins into smaller peptides (Figure 1A,B). The untreated samples displayed distinct bands in the higher molecular weight range, particularly around 50–92 kDa, as associated with legume seed storage proteins such as globulins and vicilins. For example, the ~92 kDa band observed in chickpea is likely related to the major globulin components. Following papain treatment, these high-molecular-weight bands almost completely disappeared, even at lower concentrations and shorter hydrolysis times (e.g., 2.5% papain after 1 h). This disappearance indicates the substantial cleavage of the globulin proteins into smaller peptide fragments, highlighting papain’s efficacy in hydrolyzing these complex proteins (Figure 1A).

Bromelain, while also causing visible reductions in high-molecular-weight bands, required longer hydrolysis times and higher concentrations (10% for 12 h) to achieve similar levels of protein breakdown as papain. This is in line with bromelain’s broader, but less targeted, proteolytic activity compared to papain’s specificity for bonds adjacent to arginine and lysine residues.

In contrast, ficin treatment showed partial degradation, with some higher molecular weight bands still visible even after extended hydrolysis (e.g., at 10% concentration for 12 h). The reduced effectiveness of ficin in breaking down high-molecular-weight proteins may be attributed to its narrower substrate specificity and lower activity on legume proteins, which explains the persistence of bands around 50–92 kDa.

In the lower molecular weight range (<25 kDa), new bands appeared in hydrolyzed samples, especially following papain and bromelain treatments. This pattern signifies the generation of smaller peptides as the enzymes progressively cleave the larger protein subunits. For instance, in chickpea samples treated with papain at 10%, distinct bands below 25 kDa became prominent, reflecting extensive hydrolysis. These low-molecular-weight fragments likely include oligopeptides and short-chain amino acid sequences, as observed in the SEC-HPLC analysis, which showed a peak concentration in the 200–1000 Da range (Figure 1).

Di Francesco et al. [38] demonstrated by mass spectrometry that each lane of SDS-PAGE covers the co-migration of several protein components. Mass spectrometry measurements confirmed that lipoxygenase isoforms occur with the highest relative abundance at the apparent ~90 kDa molecular weight (Mw) band of chickpea [38]. In our work, a band of ~92 kDa Mw was detected in the control (Figure 1A), which was abolished completely regardless of protease treatments. A similar trend was observed for lentils with the exception of the ficin treatment, where bands at ~92 kDa were detected using a 2.5% enzyme concentration, albeit with a lower intensity than in the control (CK).

Globulins and albumins are the most abundant protein classes in chickpea, accounting for ~56% and 18–24% of total proteins, respectively. In addition, albumins account for ~12%, while the smallest fractions are prolamins and residual proteins with 3–7% [39]. The seed storage proteins of lentils are found in the cotyledons and constitute 80% of the total protein content [40]. The most abundant storage proteins in lentils, as in other legumes, are salt-soluble globulins (70%) and water-soluble albumins (16%), while they also contain 11% glutelin and 3% prolamin [41]. Globulins make up about 50% of the entire amount of proteins of the pulse grains of the investigated both species and consist of two main groups, 11S (legumin) and 7S (vicilin) proteins [42]. In the untreated seed meal of both chickpeas and lentils, a band of ~60 kDa Mw has been identified, which is in the range of the convicilin [43]. However, Di Francesco et al. [38] revealed the co-migration of 43 proteins, of which vicilin-like proteins were the most abundant ones. Among the plant-based proteases, papain completely degraded this ~60 kDa band under the applied hydrolytical conditions, while low intensity bands were retained when conducting treatment with 2.5% ficin (Figure 1).

The 50 kDa γ-vicilin chain of 7S vicilin from the cupin superfamily is one of the earliest discovered major allergens [44]. With the addition of a 2.5% enzyme concentration, bromelain and papain hydrolyzed this chickpea polypeptide in 1 h, while in the case of ficin, the ~50 kDa band was not completely visible after 12 h of hydrolysis. The apparent ~50 kDa polypeptide of lentil proved to be more resistant to hydrolysis with bromelain than chickpea. Furthermore, a 2 h long hydrolysis duration was required for the band to be disappeared, and, even with a lower intensity, a residual band was still detected in the case of ficin.

The SDS-PAGE results underscore the differential efficiencies of the enzymes used: papain proved most effective in cleaving high-molecular-weight proteins into smaller peptides, as evidenced by the near-complete disappearance of bands in the higher molecular weight region. Bromelain showed intermediate activity, whereas ficin had the smallest impact on higher molecular weight proteins, consistent with its lower DH. The appearance of lower molecular weight bands in hydrolyzed samples corroborates the release of oligopeptides, further supporting the utility of papain and bromelain for applications requiring substantial protein breakdown in chickpea and lentil proteins.

### 3.2. Degree of Hydrolysis of Plant-Based Proteases

The DH serves as a crucial metric for evaluating the extent of protein breakdown during enzymatic hydrolysis, revealing insights into the efficiency and specificity of each enzyme used. The findings, summarized in Table 1, underscore significant variances in DH based on the type of the enzyme, its concentration, the duration of the hydrolysis, and the plant species involved. The data indicate that the degree of hydrolysis displays significant variations between chickpea and lentil seeds. Large differences were observed in DH regarding the function of the type of enzyme used, as well as the enzyme concentration and the hydrolysis time. Control samples treated with distilled water exhibited a minimal DH of 4% for chickpea and 3% for lentil seed hydrolysates. In contrast, samples treated with higher enzyme concentrations (10%) and extended hydrolysis times (12 h) showed the highest DH across all three enzymes. Notably, lentil hydrolysates consistently demonstrated a higher DH compared to chickpeas, regardless of the enzymolysis protocol. This trend is evident across all enzymes and conditions tested. Papain proved to be the most efficient enzyme, yielding the highest DH for both chickpea and lentil hydrolysis. For instance, at a 10% enzyme concentration, the DH for chickpea treated with papain was 19% after 1 h, which increased to 27.8% after 12 h. Similarly, lentil treated with the same concentration of papain exhibited a DH of 20.7% after 1 h, rising to 34.8% after 12 h. These results correlate with the changes in protein expression patterns observed on SDS PAGE, as the highest degradation of protein bands was observed in cases of papain treatments (Figure 1). The data also highlighted the substantial impact of enzyme concentration on DH. Higher enzyme concentrations (10%) resulted in more pronounced differences in the DH over the hydrolysis period. For example, the application of bromelain at a 10% concentration led to an increased DH of chickpea from 7.4% at 1 h to 20.5% at 12 h. Relative amounts of lentil hydrolysates showed an even more significant increase, from 14.8% to 31.2%, under the same conditions. While less effective than papain, ficin showed notable hydrolysis activity, particularly at the higher concentration. At a 10% concentration, the application of ficin increased the DH of chickpea from 8.8% at 1 h to 17.6% at 12 h. Lentil hydrolysates’ quantity generated with 10% ficin displayed an increase (DH has raised from 12.3% to 23.9% over the same period).

Bromelain also demonstrated a considerable hydrolytically effect, especially at higher concentrations and longer durations, but it was generally less effective than papain. Ficin showed the least hydrolytic activity among the three enzymes, though it still triggered significant hydrolysis compared to the control. Increasing the enzyme concentration from 2.5% to 10% generally resulted in a higher DH for both seed types. This trend was particularly noticeable for bromelain and papain. The increase in enzyme concentration had a more pronounced effect on lentil seeds compared to chickpea seeds. Extending the hydrolysis time from 1 h to 12 h significantly increased the DH for all enzyme treatments. The most dramatic increases were observed for the papain-treated samples, where the DH was nearly doubled over the 12 h long period. Lentil seeds consistently showed higher DH values compared to chickpea seeds under the same treatment conditions. This suggests that lentil proteins are more susceptible to enzymatic hydrolysis or that the structure of lentil proteins allows more efficient enzymatical decompositions.

**Figure 2:**
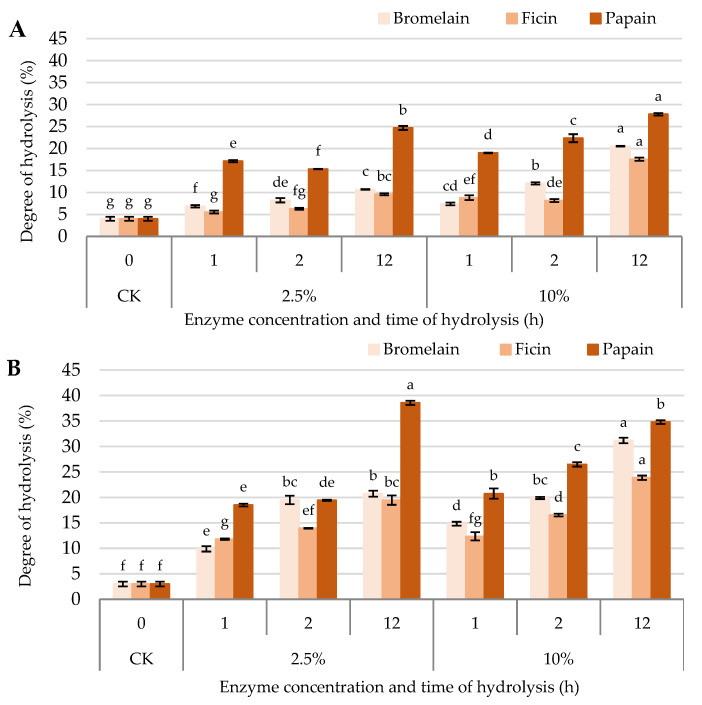
Degree of hydrolysis of (**A**) chickpeas and (**B**) lentil seeds hydrolyzed by three plant-based proteases (i.e., bromelain, ficin, and papain) at two enzyme concentrations (2.5% and 10%) for different hydrolysis times (1, 2, and 12 h). CK = control (distilled water). Different letters show significant differences at *p* < 0.05 according to Tukey’s test.

Papain consistently exhibited the highest DH values across both chickpea and lentil proteins, achieving a DH of 27.8% in chickpea and 34.8% in lentils at a 10% enzyme concentration after 12 h (Figure 2). This enhanced activity is likely attributed to papain’s strong specificity for cleaving peptide bonds adjacent to arginine and lysine residues, which are prevalent in legume proteins. Furthermore, papain’s ability to function efficiently under a wide pH and temperature range contributes to its superior hydrolytic capacity compared to other enzymes.

In comparison, bromelain demonstrated moderate hydrolytic activity, achieving a DH of 20.5% for chickpeas and 31.2% for lentils at the same concentration and duration. The moderate DH values for bromelain may stem from its broader substrate specificity, which includes peptide bonds adjacent to lysine and tyrosine residues, but with slightly lower activity against plant proteins.

Ficin, while effective, showed the least hydrolytic capacity among the three enzymes, reaching DH values of 17.6% in chickpeas and 23.9% in lentils at a 10% concentration over 12 h. Ficin’s lower efficiency may be due to its preference for specific substrates in animal rather than plant proteins, which reduces its action on the legume proteins tested here.

Each enzyme’s DH performance can be linked to its substrate specificity and catalytic mechanism. Papain’s high DH efficiency reflects its action on both globulins and albumins in legumes. Studies report that papain cleaves bonds adjacent to lysine and arginine residues, which are abundant in legume storage proteins like globulins. In SDS-PAGE analysis, papain-treated samples showed the extensive degradation of higher molecular weight proteins, indicating significant hydrolysis into smaller peptides (Figure 1).

Bromelain, known for its strong proteolytic action across various proteins, showed a relatively lower DH compared to papain. Bromelain targets lysine and tyrosine residues but is somewhat restricted by its specificity, which may explain its limited performance on legume proteins.

Ficin’s minimal DH results are likely due to its narrower substrate range, which is more effective for specific animal-derived peptides than for legume proteins. Although ficin hydrolyzes some peptide bonds, its action is comparatively slower and less effective on globulins and albumins, explaining the lower DH observed. DH increased with enzyme concentration and hydrolysis time across all enzymes tested. For instance, increasing papain concentration from 2.5% to 10% resulted in nearly double the DH for chickpea proteins over 12 h, indicating a dose-dependent effect (Figure 2). A similar trend was observed for lentil proteins, where papain achieved a DH increase from 20.7% to 34.8% under these conditions, showcasing lentil’s higher susceptibility to enzymatic breakdown. This may be due to lentil proteins’ structural attributes that allow for more efficient enzymatic access compared to chickpea proteins.

Our results corroborate findings from other studies, which indicate that the enzyme concentration and hydrolysis duration are critical factors influencing the DH of proteins. Higher enzyme concentrations and longer hydrolysis times generally enhance the breakdown of proteins, leading to a greater release of free amino groups, which is reflected in higher DH values [45,46]. Overall, the study demonstrates the varying efficiencies of different proteases in hydrolyzing chickpea and lentil proteins, with papain exhibiting superior performance. The insights gained from these results can tailor the selection of specific enzymes and can provide assistance in finding optimized conditions for protein hydrolysis in food processing and other applications.

### 3.3. Molecular Weight Distribution of Peptides in Hydrolysates

The enzymatic hydrolysis of chickpea and lentil proteins in this study resulted in a variety of smaller peptides and free amino acids, which hold the potential bioactivities and functional properties relevant to food applications. The SEC-HPLC results showed a high prevalence of peptides in the 200–1000 Da range, indicating the release of short-chain peptides, and the UHPLC analysis confirmed a significant increase in free amino acids, especially essential ones like tryptophan, arginine, leucine, and lysine.

**Figure 3:**
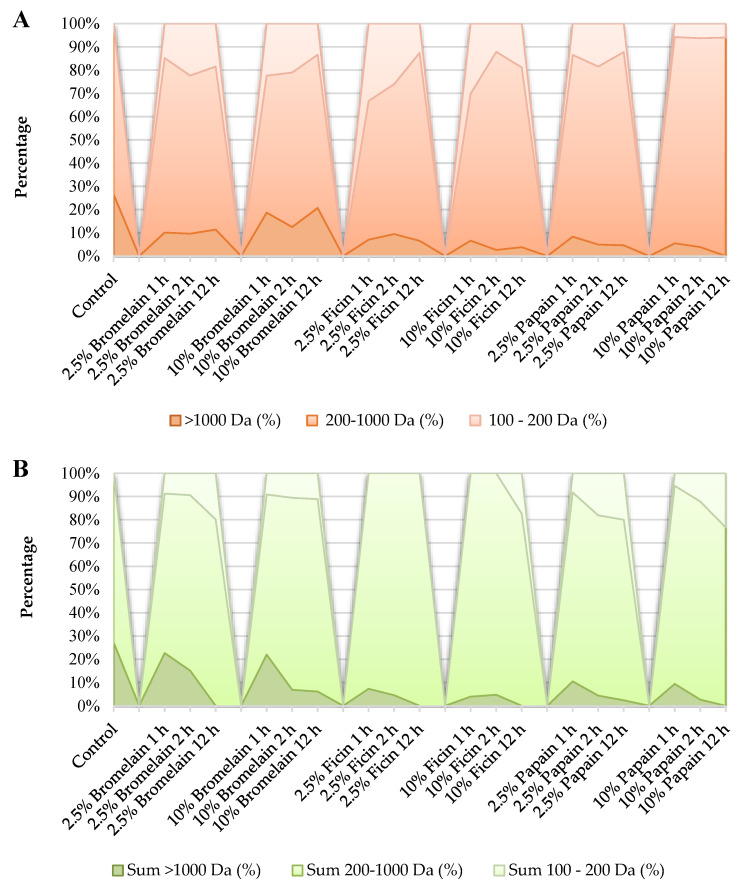
Percentages of three groups of oligopeptides/polypeptides (100–200 Da, 200–1000 Da, and >1000 Da) in hydrolysates of (**A**) chickpea and (**B**) lentil seeds that hydrolyzed by three plant-based proteases (bromelain, ficin, and papain) at two different enzyme concentrations (2.5 and 10%) for three different hydrolysis times (1, 2, and 12 h). CK = control.

Depending on the enzyme used and the duration of hydrolysis, SEC chromatography was suitable for the separation of a large number of distinctive peptides. The results, with regard to the separated polypeptides and oligopeptides in the molecular weight range below 10 kDa by SEC chromatography, generated by the hydrolysis of chickpea and lentil seeds, are summarized in Table 1 and Table 2. Based on the separable peaks, Table 1 and Table 2 indicate that a relatively small number of peptides were released in the range of 1–10 kDa as a result of enzymatic hydrolysis, and their concentration was low. In the case of chickpea hydrolysates, the ratio of peptides in the range of 1–10 kDa varied from 0 to 20.7%, while for lentil hydrolysates, peptides in the range of 1–10 kDa varied from 0 to 22.7% depending on the enzyme type, enzyme concentration, and hydrolysis time (Figure 3).

Most of the peptides were detected in the range of 200–1000 Da. Considering the concentration of total SEC-separated peptides of the chickpea hydrolysates, 55.5–93.98% of them belonged to the range of 200–1000 Da. Although the highest percentage was calculated for the papain hydrolysates, the concentration of peptides in the 200–1000 Da range was observed to be higher for the bromelain hydrolysates. For the lentil hydrolysates, 60.5–100% of the peptides appeared in the 200–1000 Da range (Figure 3). The application of ficin enzyme resulted in the formation of the highest proportion of lentil peptides in the range of 200–1000 Da, but it should be evaluated together with Table 1, from which it can be concluded that a small number of peaks could be separated with low concentrations. According to the standards, oligopeptides containing 2 to 7 amino acid units are in the range of 200–1000 Da. Protein hydrolysates containing di- and tripeptides may be advantageous from a digestive point of view because they are absorbed more rapidly than free amino acids [47]. Also, the released peptides may have bioactive properties with potential health benefits. According to Rezvankhah et al. [48], the enzymatic hydrolysis of lentil proteins can liberate bioactive peptides with plausible antioxidant activity and ACE-inhibitory properties. When the enzymatic hydrolysis of bromelain, ficin, and papain were compared, overlapping peaks were revealed in the chromatograms, but the calculation based on the standards showed that the apparent molecular weights of the peptides represented by each peak were not the same. For instance, in chickpea hydrolysates formed by using bromelain, the MW of the individual oligopeptides belonging to the highest concentration peaks fluctuated between 317 and 327 Da for 1, 2, and 12 h of 2.5% and 10% treatments. As a result of the same treatments, the MW of peptides representing individual peaks in lentil hydrolysates ranged from 305 to 319 Da. Additionally, a few peaks exhibited MWs of 100–200 Da, which, considering the average MW of amino acids (110 Da), are likely to be free amino acids or dipeptides. Typically, a small peptide or free amino acid with a size of 164–167 Da could be found in bromelain hydrolysates of both chickpeas and lentils, but no strong correlation was observed between its concentration and the treatments’ conditions.

The peptides generated through papain and bromelain hydrolysis are known for their bioactive potential, which can contribute to antioxidant, anti-inflammatory, and antihypertensive properties. Studies have shown that peptides with molecular weights below 1000 Da are particularly effective as bioactive agents due to their ability to interact more easily with biological targets, thus offering potential health benefits when included in food products [48]. The release of di- and tripeptides, such as those detected in the 200–1000 Da range, has been associated with antioxidant capabilities, which are valuable for protecting cells from oxidative damage. Bioactive peptides derived from legumes, in particular, are known to scavenge free radicals and inhibit lipid oxidation. Such antioxidant properties could improve the shelf-life and nutritional value of foods when used as natural additives [49]. Some of the oligopeptides produced, particularly those containing arginine, can exert ACE (angiotensin-converting enzyme) inhibitory effects, potentially reducing blood pressure. This activity could make these peptides suitable for functional foods aimed at promoting cardiovascular health [50].

### 3.4. Free Amino Acid Content in Protein Hydrolysates

**Figure 4:**
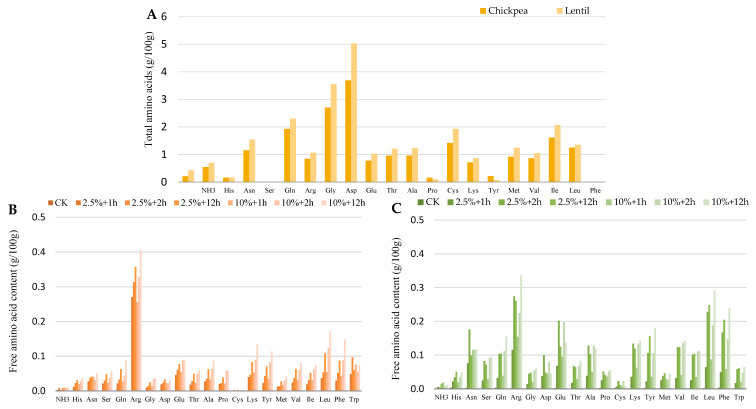

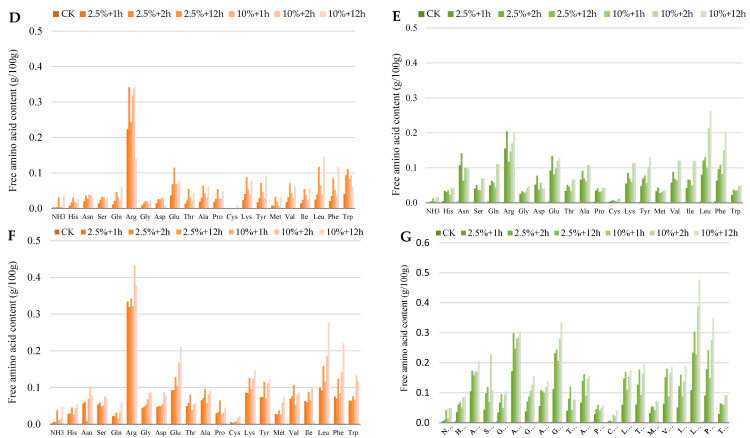
Amino acid compositions of untreated and partially hydrolyzed chickpea and lentil flour. (**A**) total amino acid composition of untreated chickpea and lentil flour; (**B**) free amino acid composition of chickpea flour hydrolyzed by bromelain, at two different enzyme concentrations (2.5 and 10%) for three different hydrolysis times (1, 2, and 12 h); (**C**) free amino acid composition of lentil flour hydrolyzed by bromelain, at two different enzyme concentrations (2.5 and 10%) for three different hydrolysis times (1, 2, and 12 h); (**D**) free amino acid composition of chickpea flour hydrolyzed by ficin, at two different enzyme concentrations (2.5 and 10%) for three different hydrolysis times (1, 2, and 12 h); (**E**) free amino acid composition of lentil flour hydrolyzed by ficin, at two different enzyme concentrations (2.5 and 10%) for three different hydrolysis times (1, 2, and 12 h); (**F**) free amino acid composition of chickpea flour hydrolyzed by papain, at two different enzyme concentrations (2.5 and 10%) for three different hydrolysis times (1, 2, and 12 h); (**G**) free amino acid composition of lentil flour hydrolyzed by bromelain, at two different enzyme concentrations (2.5 and 10%) for three different hydrolysis times (1, 2, and 12 h).

Irrespective of the enzyme type (bromelain, ficin, or papain), the enzyme concentration (2.5% or 10%), or the duration of the hydrolysis (1, 2 or 12 h), the total free amino acid (AA) contents of the hydrolysates were significantly increased in cases of both chickpeas and lentils compared to the control (Figure 4). For instance, the total AA content of the untreated chickpea (control) was found to be 21.1054 g/100 g, of which the total free AA was 0.0197 g/100 g. Enzymatic hydrolysis increased the total free AA to values between 0.5527 and 2.3331 g/100 g. The increase was at least 28-fold and up to 118-fold. The total AA content of the untreated lentils (control) was detected to be 26.9273 g/100 g, of which the total free AA was 0.0372 g/100 g. After hydrolysis, the total free AA content ranged from 0.7540 to 3.5503 g/100 g, with at least a 20-fold and up to 95.4-fold increase, depending on the enzymatic treatment. Typically, the application of the 10% enzyme during the 12 h long hydrolysis resulted in the highest amount of free AA. Of the AA released by enzymolysis, arginine (Arg) was found in the highest concentration in chickpeas, regardless of the enzyme type. However, it is typical that the use of a 10% enzyme concentration and longer hydrolysis times markedly increased the amount of free Arg concentration (Figure 4B,D,F). According to Cortes-Giraldo et al. [51], several legumes, especially chickpeas, accumulate high amounts of free Arg. Indeed, we found that arginine represented 53% of the total free AA in chickpea. In agreement with Cortes-Giraldo et al., we also found that Arg is the most abundant free AA in the chickpea flour used as control. However, it represented only 14% of the total free AA, and its concentration in the untreated chickpea flour was 0.00283 g/100 g.

Partial enzymatic hydrolysis, meanwhile, increased the amount of free Arg by at least an order of magnitude and varied between 0.2424 and 0.4311 g/100 g, depending on the conditions of the treatment. According to Sharma et al. [52], papain cuts next to Lys or Arg in addition must be flanked by a hydrophobic AA like Ala, Tyr, Trp, Val, Leu, Ile, or Phe. Along with this argument, bromelain cleaves next to Lys, Tyr, or Ala, irrespective of the flanking sequences. In this work, no marked differences were revealed in the release of Arg during hydrolysis performed with bromelain and papain. Enzyme concentration and hydrolysis time proved to be more influential factors. The availability of free arginine has been associated with a number of physiological and pathological processes; hence, its use for pharmaceutical purposes is of increasing interest [53]. The essential Arg, Leu, and Lys Aas were observed in the highest amounts in cases of both legume species formed as a result of the hydrolysis. Indeed, in the case of lentils, the hydrophobic Leu was detected at similar or higher concentrations in comparison with Arg using bromelain, ficin, and papain, subsequent to the implemented enzymolysis (Figure 4C,E,F).

Legumes are rich sources of Lys as an essential and limiting AA in several monogastric animals [54]. In chickpeas and lentils, protein-bound total Lys was quantified at concentrations of 1.42 and 1.93 g/100 g, respectively (Figure 4A). Along with it, the free Lys form was below the detection limit. However, partial hydrolysis resulted in the release of considerable amounts of protein-bound Lys. In particular, papain treatment led to a significant increase in the free Lys content in lentils, ranging between 0.0605 and 0.173 g/100 g (Figure 4G). In the case of untreated chickpeas, the free form of Trp was below the detection limit, whereas in hydrolyzed samples, its concentration ranged from 0.0485 to 0.1326 g/100 g. Among the enzymes, papain was the most effective in releasing free AAs during the hydrolysis of both chickpea and lentil proteins. As in chickpeas, Trp was below the detection limit in control lentil samples. However, the partial enzymatic hydrolysis resulted in a concentration of Trp varying between 0.0171 and 0.0922 g/100 g in the hydrolyzed samples. Lys and Gln were detected in higher amounts after this step. Trp is an essential plant-derived AA metabolically transformed to bioactive metabolites, including serotonin, melatonin, kynurenine, and the vitamin niacin (nicotinamide). Along with it, Trp is widely used as a dietary supplement for its perceived physiological benefits, including sleep and mood regulation [55]. From such an aspect, the quantitative changes in free Trp and Trp bound in oligopeptides, which can be released by enzymatic hydrolysis, may be of much practical relevance.

The increase in free amino acids, such as arginine, lysine, leucine, and tryptophan, offers both nutritional and functional advantages, especially in developing health-promoting foods. Arginine is known for its role in nitric oxide production where arginine promotes vasodilation, supporting cardiovascular health. It also plays a role in immune function and has been associated with improved muscle protein synthesis, which is valuable in sports nutrition and for individuals with higher protein requirements [51]. Lysine and leucine are essential, with lysine being critical for collagen synthesis and immune function and with leucine being known to support muscle repair and protein synthesis, which makes them particularly beneficial in protein-rich dietary supplements and functional foods targeting muscle recovery and growth [56]. Tryptophan, notably absent in untreated samples and released post-hydrolysis, plays a unique role in both health and food applications. Tryptophan is a precursor to serotonin, a neurotransmitter involved in mood regulation, sleep, and overall mental well-being. Foods enriched with tryptophan can be marketed as mood-enhancing or sleep-supporting, aligning with growing consumer interest in food products that support mental health. As an amino acid with an aromatic structure, tryptophan itself can act as an antioxidant, helping to stabilize and prevent the oxidation of other biomolecules [55]. This property could contribute to the stabilization of food products, enhancing shelf life, especially in lipid-rich foods.

Given its physiological benefits, tryptophan-enriched hydrolysates can be incorporated into functional foods, nutraceuticals, and dietary supplements that support wellness. For instance, beverages or snacks formulated with tryptophan-enriched hydrolysates could be targeted toward consumers seeking natural solutions for better sleep or mood enhancement [54]. The SEC-HPLC and UHPLC findings suggest that the peptides and amino acids released through the enzymatic hydrolysis of chickpea and lentil proteins offer bioactive and functional properties with broad applications in food. In particular, tryptophan’s unique role in supporting mood and antioxidant activity enhances the value of these hydrolysates in developing functional foods that meet consumer demands for health-promoting dietary options. These findings underscore the suitability of papain for industrial applications where high DH is desired, as it efficiently produces smaller peptides and free amino acids, potentially enhancing the bioavailability and functional properties of legume proteins. Bromelain may serve as an alternative in applications requiring moderate hydrolysis, while ficin’s role could be tailored to specialized formulations with specific hydrolysis targets.

In summary, this study highlights the distinctive mechanisms of papain, bromelain, and ficin in hydrolyzing chickpea and lentil proteins, emphasizing papain’s superior efficacy. Future research could explore the optimized conditions for each enzyme and assess the functional and bioactive properties of the resulting hydrolysates for nutraceutical and food processing applications.

The enzymatic hydrolysis of chickpea and lentil proteins in this study has produced protein hydrolysates enriched with bioactive peptides and free amino acids. These hydrolysates offer several potential applications in the development of functional foods and nutraceuticals, particularly due to their improved bioavailability, digestibility, and health-promoting properties.

The study’s findings highlight the effectiveness of papain, bromelain, and ficin in breaking down complex legume proteins into peptides and free amino acids, notably essential amino acids like tryptophan, arginine, and lysine. These hydrolysates, due to their rich amino acid profiles, can be incorporated into plant-based foods to enhance their nutritional value, particularly in vegan or vegetarian products where meeting amino acid requirements can be challenging. This makes chickpea and lentil hydrolysates valuable as protein supplements or ingredients in high-protein foods, such as plant-based protein bars, beverages, and meal replacements. The bioactive peptides released, especially those in the 200–1000 Da range, are associated with several health benefits: Short-chain peptides and free amino acids like tryptophan can neutralize free radicals, providing antioxidant protection. This makes these hydrolysates suitable for functional foods aimed at reducing oxidative stress, which is linked to aging and various chronic diseases. Peptides containing arginine contribute to nitric oxide production, which supports vasodilation and cardiovascular health. This feature is valuable for functional foods targeted at heart health, a growing consumer market segment. Tryptophan, a precursor to serotonin, can aid in mood regulation and sleep quality, making these hydrolysates suitable for nutraceuticals or functional foods focused on mental well-being. The development of tryptophan-enriched snacks or supplements could appeal to consumers seeking natural solutions for improved mood and sleep. Partially hydrolyzed proteins are more easily digested and absorbed, which can benefit individuals with digestive sensitivities or those requiring easy-to-digest protein sources, such as athletes or older adults. By incorporating these hydrolysates into food products, manufacturers can create high-protein foods that are gentle on the digestive system, catering to a broad consumer base with specialized dietary needs. The antioxidant properties of certain peptides and free amino acids, such as tryptophan, also have implications for product stability. Their addition could improve the shelf-life of functional foods, especially those with higher fat contents, by preventing lipid oxidation. This can enhance product quality and freshness, providing a natural alternative to synthetic antioxidants in the food industry. Given the bioactive potential of chickpea and lentil hydrolysates, they hold promise for nutraceuticals designed for specific health benefits, such as muscle recovery (via leucine and arginine content) or cardiovascular support. Such hydrolysates can be marketed as dietary supplements, capsules, or powders for consumers interested in targeted health outcomes. The study’s insights into the enzymatic hydrolysis of legume proteins align with the growing demand for plant-based functional foods and nutraceuticals. The ease of incorporating chickpea and lentil hydrolysates into diverse product forms—whether in beverages, bars, or supplements—demonstrates their versatility and appeal for modern dietary trends focusing on health, sustainability, and convenience. As the consumer awareness of food’s role in promoting well-being grows, chickpea and lentil protein hydrolysates offer an innovative pathway for creating nutrient-dense, bioactive-rich products.

Also, given its physiological benefits, tryptophan-enriched hydrolysates can be incorporated into functional foods, nutraceuticals, and dietary supplements that support wellness. For instance, beverages or snacks formulated with tryptophan-enriched hydrolysates could be targeted toward consumers seeking natural solutions for better sleep or mood enhancement [54]. The SEC-HPLC and UHPLC findings suggest that the peptides and amino acids released through the enzymatic hydrolysis of chickpea and lentil proteins offer bioactive and functional properties with broad applications in food. In particular, tryptophan’s unique role in supporting mood and antioxidant activity enhances the value of these hydrolysates in developing functional foods that meet consumer demands for health-promoting dietary options. These findings underscore the suitability of papain for industrial applications where a high DH is desired, as it efficiently produces smaller peptides and free amino acids, potentially enhancing the bioavailability and functional properties of legume proteins. Bromelain may serve as an alternative in applications requiring moderate hydrolysis, while ficin’s role could be tailored to specialized formulations with specific hydrolysis targets. Overall, the release of peptides and free amino acids facilitated by plant proteases can be beneficial for digestibility and absorption. Bioactive hydrolysates and peptides can also be used as functional ingredients due to their specific properties such as the modulation of gastrointestinal absorption, appetite suppression, opioids, immunomodulation, etc., and therefore may be beneficial as ingredients in food formulations [57]. Bioactive peptides are short amino acid sequences, typically consisting of 2–30 amino acids, with a simple structure in the low-molecular-weight range. And our present experiment confirmed that both the relative proportion and absolute concentration of peptides in this low-molecular-weight range of 200–1000 Da were significantly increased in both plant species studied, especially with the hydrolysis of bromelain and papain. However, the bioactive properties of the peptide mixtures and fractionable individual peptides we obtained need to be investigated in detail. Once proven to have biological activity, food peptides become natural ingredients in functional food formulations. Food peptides are potential therapeutic agents due to their remarkable biospecificity, activity, spectrum of activity, and structural diversity. In addition to their remarkable biospecificity and therapeutic effects, food peptides can also be beneficial from a food safety perspective, as they prevent oxidation and microbial spoilage [58,59]. However, the scaling up of bioactive peptides still has limitations and challenges, including, e.g., the taste and organoleptic quality of the product and potential allergenicity, which are important factors.

## 4. Conclusions

Our study provides a detailed examination of the hydrolysis of chickpea and lentil proteins using three plant-based proteases: bromelain, ficin, and papain, at two enzyme concentrations (2.5% and 10%) and three hydrolysis durations (1, 2, and 12 h). Papain exhibited the highest hydrolytical efficiency in cases of both legumes, with a DH of 27.8% for chickpeas and of 34.8% for lentils after 12 h long hydrolysis at a 10% enzyme concentration. Consistently higher DH values were observed for lentil proteins compared to those of chickpeas under all conditions, suggesting the higher susceptibility of lentils to enzymatic breakdown. SDS-PAGE analysis revealed significant protein degradations across all treatments, with the most pronounced effects being observed with papain and bromelain. Notably, papain completely degraded the ~92 kDa band in chickpeas and significantly reduced it in lentils, indicating the effective cleavage of high-molecular-weight proteins. SEC-HPLC showed that peptides in the range of 200–1000 Da were the most prevalent, particularly in the case of papain treatment. Arg was detected to be the most abundant AA acid released, particularly under papain treatment, aligning with its known cleavage specificity. The study focused on three plant-based proteases; however, there are many other proteases whose effectiveness could be involved in a comparative study. The mechanisms of enzyme specificity were not deeply explored, limiting the understanding of distinct features of certain enzymes’ performances. The study did not include functional or bioactivity assays to evaluate the potential health benefits of the hydrolysates. Apart from possible limitations of this work, our research contributed a more comprehensive interpretation of three distinct plant-based proteases, as well as providing food manufacturers and food technologist with relevant pieces of information regarding novel manners to improve the composition and bioutilization of protein rich products. The modification of the amino acid profiles and the enhancement of the oligopeptide content of two well-known basic materials (lentil and chickpea) via the enzymatical hydrolysis of plant proteins may lead to the elaboration of functional foods and nutraceuticals of improved nutritional and biological value.

## Figures and Tables

**Table 1:** Concentration of oligopeptides/polypeptides identified in hydrolysates of chickpea seeds that hydrolyzed by three plant-based proteases (bromelain, ficin, and papain) at two different enzyme concentrations (2.5 and 10%) for three different hydrolysis times (1, 2, and 12 h).

		**Bromelain**											
Control		2.5%						10%					
		1 h		2 h		12 h		1 h		2 h		12 h	
MW ^†^ (Da)	Conc. (mg/g)	MW (Da)	Conc. (mg/g)	MW (Da)	Conc. (mg/g)	MW (Da)	Conc. (mg/g)	MW (Da)	Conc. (mg/g)	MW (Da)	Conc. (mg/g)	MW (Da)	Conc. (mg/g)
3317	7.039	6466	3.2	3371	8.2	3161	4.1	3289	6.3	3371	8.7	3210	3.5
1730	6.129	3229	9.4	1679	4.2	1696	3.4	1667	9.0	1681	12.6	1675	14.7
1091	4.490	904	18.8	1073	6.1	1079	2.2	1091	9.0	1101	3.4	1078	12.0
872	6.978	635	12.3	897	14.3	840	4.6	896	17.5	860	11.4	835	14.9
664	6.432	486	29.5	639	11.7	651	6.3	646	9.1	643	8.9	648	11.8
500	18.022	406	20.6	487	25.1	497	14.3	488	15.0	487	26.0	490	19.1
314	17.415	329	12.1	397	30.2	325	34.3	399	14.3	398	31.3	317	50.1
		165	18.4	327	49.4	168	15.7	325	20.3	323	53.9	167	19.4
				164	43.0			165	29.1	164	41.7		
		**Ficin**											
		2.5%						10%					
		1 h		2 h		12 h		1 h		2 h		12 h	
		MW (Da)	Conc. (mg/g)	MW (Da)	Conc. (mg/g)	MW (Da)	Conc. (mg/g)	MW (Da)	Conc. (mg/g)	MW (Da)	Conc. (mg/g)	MW (Da)	Conc. (mg/g)
		3247	7.6	3308	8.3	3260	5.8	3377	8.9	3084	9.8	2967	4.7
		912	10.6	875	17.7	844	4.7	926	14.1	905	73.8	670	10.1
		649	8.3	481	15.6	659	10.3	663	7.6	787	59.8	501	27.8
		484	15.9	323	23.1	492	25.1	502	19.4	489	33.4	412	15.3
		398	13.0	160	22.8	309	19.8	410	15.5	396	30.3	321	42.8
		329	24.9			260	11.9	336	28.1	311	70.1	178	23.4
		164	35.8			180	11.2	170	40.5	266	48.5		
										162	45.0		
		**Papain**											
		2.5%						10%					
		1 h		2 h		12 h		1 h		2 h		12 h	
		MW (Da)	Conc. (mg/g)	MW (Da)	Conc. (mg/g)	MW (Da)	Conc. (mg/g)	MW (Da)	Conc. (mg/g)	MW (Da)	Conc. (mg/g)	MW (Da)	Conc. (mg/g)
		3096	9.8	3123	9.3	3080	5.2	3177	7.3	3116	5.3	625	35.7
		859	6.8	871	23.5	629	8.6	879	10.2	1088	14.3	463	47.9
		608	9.9	610	9.2	464	27.2	620	9.9	877	13.0	293	66.2
		452	28.0	449	33.6	296	57.9	463	29.1	618	12.2	184	9.6
		286	47.1	295	77.7	191	13.8	293	67.9	462	26.6		
		178	15.8	161	34.8			186	7.6	293	56.0		
										188	8.6		

^†^ Molecular weight.

**Table 2:** Concentration of oligopeptides/polypeptides identified in hydrolysates of lentil seeds that hydrolyzed by three plant-based proteases (bromelain, ficin, and papain) at two different enzyme concentrations (2.5 and 10%) for three different hydrolysis times (1, 2, and 12 h).

		**Bromelain**											
Control		2.5%						10%					
		1 h		2 h		12 h		1 h		2 h		12 h	
MW ^†^ (Da)	Conc. (mg/g)	MW (Da)	Conc. (mg/g)	MW (Da)	Conc. (mg/g)	MW (Da)	Conc. (mg/g)	MW (Da)	Conc. (mg/g)	MW (Da)	Conc. (mg/g)	MW (Da)	Conc. (mg/g)
7125	7.949	6708	3.0	3179	7.8	730	35.0	6574	2.2	1688	29.4	1696	24.0
3179	3.277	3128	4.2	1701	20.2	490	30.4	3186	5.2	907	64.3	719	53.5
1984	3.155	1779	15.8	1075	41.4	397	50.4	1701	25.7	722	57.8	487	51.1
739	15.109	1072	16.7	919	67.5	313	104.1	1078	23.3	484	43.8	393	69.1
490	7.100	926	24.2	721	55.5	174	54.3	828	25.8	398	67.1	310	150.5
245	16.201	731	23.0	487	39.4			729	30.3	314	78.4	168	43.6
		490	12.2	394	49.0			490	15.9	253	41.0		
		398	15.8	305	88.9			399	31.9	165	44.8		
		309	22.4	250	46.5			319	43.0				
		248	22.8	164	43.2			250	29.9				
		166	15.4					166	23.5				
		**Ficin**											
		2.5%						10%					
		1 h		2 h		12 h		1 h		2 h		12 h	
		MW (Da)	Conc. (mg/g)	MW (Da)	Conc. (mg/g)	MW (Da)	Conc. (mg/g)	MW (Da)	Conc. (mg/g)	MW (Da)	Conc. (mg/g)	MW (Da)	Conc. (mg/g)
		3031	4.4	2970	2.8	686	75.2	2970	2.8	3177	3.2	910	57.9
		774	16.1	775	22.2	275	57.2	761	17.5	743	17.7	707	66.9
		270	39.4	272	36.5			275	50.5	276	47.1	403	78.8
												334	81.3
												293	98.0
												248	83.1
												174	98.1
		**Papain**											
		2.5%						10%					
		1 h		2 h		12 h		1 h		2 h		12 h	
		MW (Da)	Conc. (mg/g)	MW (Da)	Conc. (mg/g)	MW (Da)	Conc. (mg/g)	MW (Da)	Conc. (mg/g)	MW (Da)	Conc. (mg/g)	MW (Da)	Conc. (mg/g)
		6943	2.6	7212	1.8	1910	8.1	6967	3.4	3084	9.8	741	46.9
		3272	9.8	3132	4.2	746	35.6	3186	8.3	905	73.8	410	70.4
		1903	8.3	919	38.8	501	29.4	1910	6.9	787	59.8	326	121.7
		967	22.7	789	32.2	413	57.8	963	29.9	489	33.4	171	72.8
		853	21.7	496	9.8	328	95.4	838	25.5	396	30.3		
		752	24.5	401	5.6	264	45.4	753	27.6	311	70.1		
		508	13.5	317	26.2	173	68.0	508	11.7	266	48.5		
		414	12.0	266	33.4			315	36.7	162	45.0		
		317	32.9	164	25.1			264	36.8				
		263	33.1					171	10.6				
		170	16.3										

^†^ Molecular weight.

## Data Availability

Data will be made available upon the request.
